# Development of Healthy Snacks Incorporating Meal from *Tenebrio molitor* and *Alphitobius diaperinus* Using 3D Printing Technology

**DOI:** 10.3390/foods13020179

**Published:** 2024-01-05

**Authors:** Francisco Madail Herdeiro, Maria Otília Carvalho, Maria Cristiana Nunes, Anabela Raymundo

**Affiliations:** LEAF—Linking Landscape, Environment, Agriculture and Food Research Center, Associate Laboratory TERRA, Instituto Superior de Agronomia, Universidade de Lisboa, Tapada da Ajuda, 1349-017 Lisbon, Portugal; fherdeiro@isa.ulisboa.pt (F.M.H.); motiliac@isa.ulisboa.pt (M.O.C.); cristiananunes@isa.ulisboa.pt (M.C.N.)

**Keywords:** 3D food printing, edible insects, *Tenebrio molitor*, *Alphitobius diaperinus*, sustainability

## Abstract

This study analyzes the nutritional properties of edible insects, specifically *Tenebrio molitor* and *Alphitobius diaperinus*, and explores the potential of 3D printing technology to introduce a nutritious and tasty alternative to essential nutrients for Western consumers. An original formulation for the printing of snacks with microalgae was adapted to incorporate edible insects. Concentrations of 10% of edible insects, both isolated and mixed, were incorporated into the developed ink-doughs. Stress and frequency sweeps were performed on the doughs to understand the rheology and the impact on the internal structure to better adapt these materials to the 3D printing process. The nutritional profile of the developed snacks was assessed, revealing a significant amount of protein, enough to claim the snacks as a “source of protein”, as well as an increased mineral profile, when compared to the control snack. The antioxidant profile and total phenolic content were equally assessed. Finally, a sensory analysis test was performed, comparing the control snack to three other samples containing 10% *T. molitor*, 10% *A. diaperinus* and 5% + 5% of *T. molitor* and *A. diaperinus*, respectively, resulting in a preference for the *A. diaperinus* and for the combination of the two insects. Considered as a “novel food”, foods incorporating edible insects represent, in fact, the reintroduction of foods used in the West before the Middle Ages, when the Judeo-Christian tradition began to consider insects as not kosher. Educating consumers about the transition to novel foods can be helped by 3D printing food, as an innovative process that can be used to design creative rich animal protein snacks that make final products more appealing and acceptable to consumers.

## 1. Introduction

With the expectation that the global population will reach close to 10 billion by 2050 [1], concerns are growing about the availability of agricultural land and scarcity of animal protein, given the difficulty that global food systems will face in keeping up with such growth [2] if alternatives are not found.

The prices of the main sources of animal protein have risen over the years and are expected to continue to rise, leaving some segments of society unable to purchase and consume animal protein as well as other nutrients essential to human development like group B vitamins. To avoid a total lack of protein in diets, resulting from food insecurity, alternatives have been studied for food (insects, fungi, cultured meat, micro and macroalgae) and for feed (insects, food waste, biofuel by-products) that are potentially nutritionally healthy and environmentally sustainable [3,4,5].

Historically, entomophagy has been associated with human diet and evolution, in many different cultures and regions of the world [5,6]. According to estimates, more than 2000 insect species are consumed in about 140 countries [7], mainly in Asia, Australia, Africa and in the Americas [8,9,10,11,12,13]. Europe also had entomophagy habits, although this diet was only available during warm seasons. This availability worsened between the XII and XVIII centuries due to this region suffering from “The Small Ice Age”, together with the spreading of the Abrahamic religions that considered insects as “dirty” food for human consumption [6,14]. FAO has encouraged more entomophagy in the world and Europeans, with the help of the EU, are starting to accept the benefits of entomophagy and to include them in human diets [8].

The mealworm beetle, *Tenebrio molitor* L. (Coleoptera, Tenebrionidae), is a worldwide phytophagous insect associated with stored cereals. The larva of *T. molitor* is also associated with stored cereals [15,16].

The lesser mealworm beetle, *Alphitobius diaperinus* Panzer (Coleoptera, Tenebrionidae), is a pest of stored cereals, and derivatives, as well as poultry livestock sheds, manifesting at ground level in animal bedding [17]. *A. diaperinus* feeds in their feed and the larvae attack wood, fiberglass and polystyrene insulation to pupate [18]. This species was nominated as an edible insect and has been introduced to the market as a source of protein for fish feed in aquaculture [19,20] and later for animal feed. *A. diaperinus* can have a higher protein content than *T. molitor* [19]. Several studies report that edible insects can reach between 40 and 70% protein in dry weight, when compared to other protein sources, like meat, dairy or seeds [21]. However, Kurečka et al. [22] found otherwise, with *T. molitor* having a more significant percentage of protein, with the proviso that this difference from other authors might come from differences in feed substrates. *A. diaperinus*, by being a pest [18], has a faster reproductive cycle than that of *T. molitor*, which contributes to the reduction in production costs in terms of yield per mass, becoming a more affordable and nutritious option for the consumer, both for food and feed [22].

Edible insects can also be an important, and unexpected, source of bioactive compounds, including phenolic compounds, tocopherols and phytosterols, as demonstrated by [23,24]. The process of absorbing these compounds occurs through the process of sclerotization, a process in which phenolic compounds are integrated into the insect epidermis through a series of enzymatic reactions [25].

The high amount of meat and cereals in diets places a high demand on the production and maintenance of resources associated with agricultural production and animal husbandry [16]. As such, insects will effectively become part of our diet due to the various factors associated with both our foodstuffs and their impacts on our health, environmental sustainability and circular economies [16]. Thus, insects appear as an alternative to replace, from the origin, animal protein, as well as carbohydrates, fat ω-3 and ω-6, and other micronutrients [25]. These insects, due to their small size, do not require extensive production areas, and because they can feed on by-products of organic matter, they do not need a specific nutrient source, which means they will be able to feed on organic waste fostering the ideal of circular economy and zero waste.

Three-dimensional printing, also known as additive manufacturing, is an innovative process that has arrived as an important tool for manufacturing food as well as an innovation in the design of novel foods and that keeps on growing in popularity both with the stakeholders of the food industry and consumers [26]. It can incorporate ingredients that can unease consumers, because of their color, smell or taste, for which microalgae [27,28,29] or edible insects [30,31] can be an example. Differentiated design has a positive impact on the perception that consumers have of alternative ingredients. This study aims to create room for edible insects to take a chance at impressing consumers, either by their taste or through the differentiated designs presented to the consumers, in this case “duck footings”.

Therefore, this work aims to study the pertinence of the use of *T. molitor* and *A. diaperinus,* which were approved as novel foods in 2021 [20] and flours in the formulation of innovative foods, increasing the diversity of supply that the sector can offer to the consumer and providing tasty, safe and sustainable alternatives. Different snack formulations were evaluated, always from the perspective of which is the most effective and efficient in terms of consumer acceptance. These different formulations were characterized in rheological, nutritional and biochemical terms. The snack designs were drawn with the help of 3D printing technology and evaluated by a sensory panel to test the acceptance of the different snacks. The study follows the BugSnack Project protocol [12], which seeks to create a portfolio of innovative, healthy and sustainable snacks in line with the trend of snackification. The preparation and analysis of control samples for the study of the formulation and characterization of insect-based snacks aimed to understand the influence of different ingredients on the properties of the ink-dough used for printing.

## 2. Materials and Methods

### 2.1. Dough Preparation and Printing

The authors used a formulation (denoted as control) from previous works [27,28] of snacks with microalgae incorporation, as the base for the samples. The formulation consisted of wheat flour (Nacional, Cerealis, Maia, Portugal), rice flour (Ceifeira, Dacsa Atlantic, Lisboa, Portugal), maize starch (Maizena, Unilever Fima, Lisboa, Portugal), xanthan gum (SOSA, Barcelona, Espanha), edible oil (Fula, Sovena, Algés, Portugal), commercial salt and distilled water (Table 1).

Variations were made by altering the proportions of these ingredients to test their influence on the dough properties during printing. Wheat flour, rice flour and maize starch were adjusted individually in separate samples. Levels of 8% and 12% insect flours were tested but did not work. The edible oil was removed from insect-containing samples once it was noticed that it complicated the 3D printing process. *Tenebrio molitor* (Portugal Bugs, Perafita, Portugal) and *Alphitobius diaperinus* (Entogreen, Santarém, Portugal) were used.

**Figure 1:**
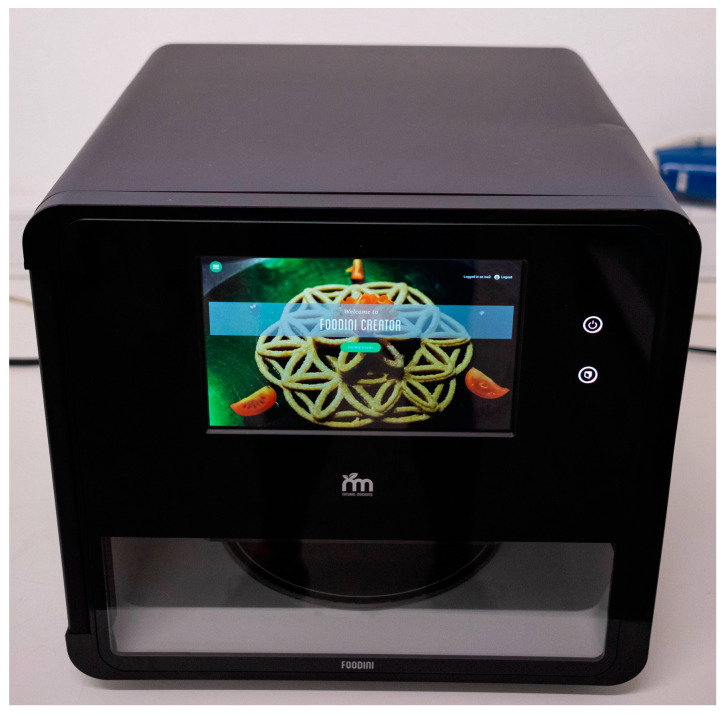
3D food printer (Foodini, Natural Machines, Barcelona, Spain).

The dough preparation process involved weighing the ingredients according to the formulations, mixing the dry ingredients by hand with the help of a spatula until the mixture became homogeneous, adding distilled water and achieving a homogeneous mixture. The dough was then left to stand for 10 min before being loaded into a syringe for printing, using a 3D printer (Foodini, Natural Machines, Barcelona, Spain) (Figure 1).

The printing process was performed by inserting 100 g of dough inside a cartridge with a 1.5 mm nozzle at a temperature of 20 ± 1 °C, with an output of 1.12 mL/min.

After printing, the snacks were transferred to a pre-heated forced-air convection oven XFT133 (Unox, Cadoneghe, Italy) at 180 ± 5 °C, to bake for 10 min. When the snacks were ready, they were transferred to a proofer (Unox, Cadoneghe, Italy) pre-heated at 70 ± 5 °C and left to rest for 10 min to ensure a slow cool down.

### 2.2. Snack Characterization 

#### 2.2.1. Dough Rheological Properties

For the assessment of the rheological properties of the doughs the measurements were based on small amplitude oscillatory shear (SAOS), using a controlled stress Haake MARS III (Thermo Fisher Scientific, Waltham, MA, USA) coupled with a TC Peltier.

Measurements were performed using a 20 mm diameter serrated plate–plate, to overcome the slip effect. After mixing and resting, the dough samples were placed in the apparatus and then were covered with liquid paraffin after achieving the measurement position, to avoid evaporation during the tests. The gap of 1 mm was adjusted (previously optimized for this type of material [27]).

Stress and frequency sweep tests were performed to analyze the dough viscoelasticity. The stress sweep tests at 1 Hz were performed to define the linear viscoelastic region (LVR). The LVRs defined for the mechanical spectra were for the control 3.9 Pa, and 7.3 Pa for the A10%, T10% and AT10% samples. The mechanical spectra were obtained by frequency sweeps which were performed at a selected stress within the LVR, and frequencies ranging from 0.1 to 50 Hz. All the measurements were performed at 20 °C, at least, in triplicates.

#### 2.2.2. Texture Properties

Penetration tests were performed to assess the texture parameters of the baked snacks, using a TA.XTplus texturometer (Stable Microsystems, Surrey, UK), with a 30 kg load cell and a 2 mm diameter cylindrical stainless steel probe, as described by Letras et al. [27] and Oliveira et al. [28]. Measurements were performed in a room with a controlled temperature of 20° ± 0.5 °C. This test allowed obtaining the hardness (N) of the snacks. Hardness was obtained from the peak force (N) in a force versus time texturogram, being the force required to break the snack [32].

#### 2.2.3. Nutritional Characterization

The nutritional characteristics of the snacks evaluated the quantity of moisture, ash, total fat, total fiber, total protein and mineral content for each sample. The moisture content, ash content, total protein and total fat content of the samples were analyzed using specific methods. The carbohydrates were assessed by difference.

For the biochemical characterization of the samples, the snacks were ground to a powder and analyzed using three replications.

The moisture content was measured by using porcelain melting pots that had been previously placed in an oven (Binder GmbH, ED056, Tuttlingen, Germany) at 104 ± 1 °C for 1 h to remove any previous moisture content, as it was carried out by [29]. The melting pots were placed in an oven with 5 g of each sample for 24 h. After 24 h the melting pots were placed in a desiccator for cooling and weighed on a digital scale (Denver Instrument Company, TC-403, Arvada, CO, USA).

For the measurement of the ash according to AACC 08-01, new samples were placed in melting pots and were placed in a muffle furnace (Snol, Utena, Lithuania) at 500 ± 1 °C for 24 h to obtain the ash. After 24 h the weight of the ash was measured.

The total protein content of the samples was obtained by preparing samples in triplicates and using DUMAS equipment (Thermo Quest NA 2100 Nitrogen and Protein Analyser, Interscience, Breda, The Netherlands), which evaluates the nitrogen content of the samples, through combustion, allowing the determination of protein content as a percentage of nitrogen times a conversion factor (6.25).

The total fat content of each sample was obtained by hydrolysis [33]. Triplicates of each formulation were prepared and added to a mixture of methanol (CH_3_OH), chloroform (CHCL_3_) and hydrochloric acid (HCl), on a proportion of 10:1:1.5, respectively. This mixture was then extracted with a mixture of hexanes (CH_3_(CH_2_)_4_CH_3_) and chloroform on a ratio of 4:1 (*v*/*v*), placed under a vortex for 2 min and processed to be centrifuged (Z383 K, Hermle-Labnet International, Edison, NJ, USA), for 10 min at room temperature at 9600 g. The fat fraction of the now separated mixture was removed into glass tubes that had been previously weighed. The tubes were placed inside an oven at 50 ± 5 °C, for a duration of 3 days and subsequently weighed. The difference between the initial and final weight of the tubes is the total fatty acid content of the samples.

The total carbohydrate content of the samples was determined by difference and energy (kcal/100 g) was determined through the conversion factors indicated by Annex XIV of Regulation (EU) No. 1169/2011 [34].

The mineral profile (Na, K, Ca, Mg, P, S, Fe, Cu, Zn, Mn and B) of each sample was determined in triplicates of 500 mg, using inductively coupled plasma optical-emission spectrometry (5800 ICP-EOS, Waltham, MA, USA—Thermo ScientificTM iCap Serires 7000, Thermo Fisher Scientific, Waltham, MA, USA) [35,36]. An aid digestion was performed through the addition of 12 mL of hydrochloric acid (HCl) and 4 mL of nitric acid (HNO_3_) on a ratio of 3:1 to each sample. A cool down time of 24 h was given to the mixtures and upon reaching room temperature, they were filtered and diluted to 50 mL with distilled water.

The Dosi-Fiber method was used to determine the crude fiber content in the samples. To perform the method, a fine powder of 1 to 1.5 g of the sample was weighed into a glass crucible with a filtering bottom. The crucibles were heated with a sulfuric acid solution, then with a potassium hydroxide solution. Afterwards, a cold extraction with acetone was performed, and the crucibles were washed and dried before being heated in a muffle furnace [35].

### 2.3. Antioxidant Capacity

The antioxidant potential of the snacks was assessed by the ferric reducing antioxidant power (FRAP) and 2,2-Diphenyl-1-picrylhydrazyl (DPPH) methods. An initial extraction was performed: firstly 4 g of each sample (control, T10%, A10% and AT10%) was turned into a powder and dissolved in 8 mL of ethanol (96%). Immediately after, the samples were centrifuged for 10 min at 9600× *g*. The extracts were then filtered through a 0.2 μm syringe (B. Braun Medical Lda., Barcarena, Portugal) connected to filters (NY) and the ethanol was evaporated under vacuum by means of a rotatory evaporator (N-490, Buchi Ibérica, Barcelona, Portugal). The dried extracts were re-dissolved in 20 g of dimethyl sulfoxide (DMSO) (C_2_H_6_OS), resulting in stock solutions at concentrations of 20 mg/mL that were properly stored at 4 °C until the moment of determination of antioxidant activity and total phenolic content.

#### 2.3.1. DPPH

For the 2,2-Diphenyl-1-picrylhydrazyl (DPPH) assay, the previous extracts were used. A calibration curve was generated using ascorbic acid, diluted from a stock solution (1 mg/mL) with distilled water to various concentrations (0, 10, 25, 50, 75, 100, 150, 200 and 250 μg/mL) [19,20]. Triplicates of each standard solution were prepared by adding 3.9 mL of DPPH (C_18_H_12_N_5_O_6_) (60 µmol/L) to 0.1 mL of each dilution and incubating for 1 h in the dark. After the incubation was finished, methanol (CH_3_OH) was used as a blank for the spectrophotometer (Agilent Technologies, Cary 60 UV-Vis, Santa Clara, CA, USA). This way the calibration curve was obtained at 515 nm [19,20]. The negative control was performed by replacing the extract with water [27,35,37].

#### 2.3.2. FRAP

For the determination of the antioxidant activity by the ferric reducing antioxidant power (FRAP) assay method, triplicates of each sample were prepared. Alongside this, several solutions were also prepared to produce the intended reaction: a 40 mM HCl solution, a 2,4,6-Tris(2-pyridyl)-s-triazine (TPTZ) (C_18_H_12_N_6_) solution, a ferric chloride (FeCl3) solution and an acetate (0.3 M) buffer (pH = 3.6). The FRAP reagent was obtained by mixing the solutions of TPTZ, ferric chloride and the sodium acetate buffer in a proportion of 1:1:10, respectively [27,35]. In addition, to obtain a calibration curve, several dilutions (0, 10, 25, 50 and 75 μg/mL) of an ascorbic acid stock solution (1 mg/mL), using distilled water, were made.

An amount of 90 μL of each respective ascorbic acid dilution was pipetted, to which 270 μL of distilled water and 2.7 mL of FRAP reagent were added. The solutions were then homogenized and incubated in a water bath (Thermo Scientific, 2871, Waltham, MA, USA), at 37 °C for 30 min. Each replication (3) sample extract was prepared by adding 90 μL of each extract combined with 270 μL of distilled water and 2.7 mL of FRAP solution, homogenized using a vortex and incubated in a water bath under the same conditions. After 30 min incubation time, the absorbances of these solutions were read at 595 nm with distilled water used as a blank. To calculate the ascorbic acid equivalent values from the absorbance values, the calibration curve parameters obtained from their linear regression were used [27,38].

#### 2.3.3. Total Phenolic Content

The total phenolic compounds of the sample extracts were assessed by combining 150 μL of sample extract with 140 μL of a Folin–Ciocalteu solution (12%) and 2.4 mL of distilled water. After homogenizing the solution in a vortex, it was left to rest for 3 min before adding 300 μL of sodium carbonate (Na_2_CO_3_) (10%). All the tubes were left to incubate in a dark environment at room temperature for a 2 h period. After the incubation, absorbances were read at a 725 nm wavelength, using distilled water as a blank. Results were expressed as gallic acid (C_6_H_2_(OH)_3_CO_2_H) equivalents (mg GAE) per g of dry weight [27,39].

### 2.4. Opinion Study

An opinion study was performed with a panel composed of 34 individuals, from both genders, with ages ranging between <18 and 65 years old (yo) (4 <18 yo; 15 18–25 yo; 9 26–35 yo; 4 36–50 yo; 2 51–65 yo), and with education levels ranging from high school level to PhD level (13 high school, 6 bachelor, 7 masters, 5 PhDs and 3 other education levels). The consumers were first asked about their opinion on the utilization of insects in human food, and then proceeded to taste the four different snacks elaborated in this study: the control, A10%, T10% and AT10%. After the tasting, the panel was asked to review their opinion on the utilization of insects in human food, and change the mark given previously if they wanted.

### 2.5. Statistical Analysis

The one-way ANOVA was applied to the analysis of variance of the samples, as well as a Tukey Test to identify statistical significance between the different characteristics of the samples with a significance level of 95% (*p* < 0.05). The GraphPad Prism 5.0 statistical analysis software was used for this part.

## 3. Results

### 3.1. Snack Printing

**Figure 2:**
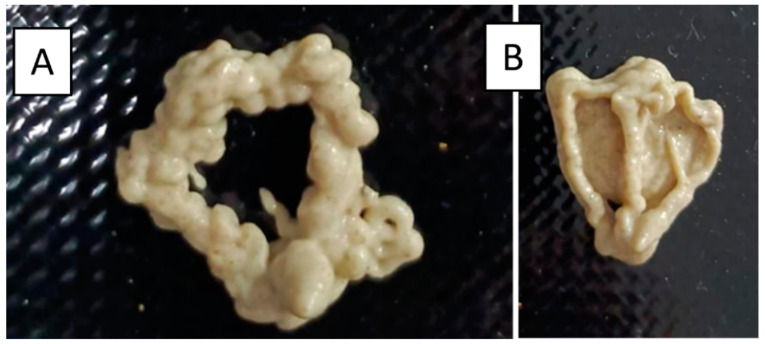
Failed snack printings of the *Tenebrio molitor* 8% (**A**) and *Tenebrio molitor* 12% (**B**) samples.

**Figure 3:**
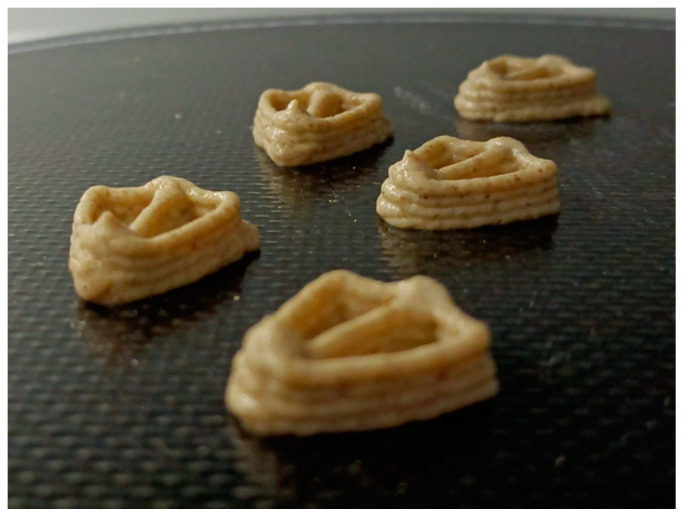
Successful snack printings of the *Tenebrio molitor* and *Alphitobius diaperinus* 10% (AT10%, 10% of *A. diaperinus* + *T. molitor*) sample.

The incorporation of insect flour had a noticeable impact on the dough flow during the printing process. Different concentrations (8, 10 and 12%) of insect flour were used as preliminary tests on the 3D printer, which resulted in varied performances, ranging from excess dough deposition to the printer’s inability to produce snacks (Figure 2 vs. Figure 3). Rheology tests (non-presented results) confirmed that formulations containing 10% of insect flour had a stronger structure compared to the control sample. The difference in development could be attributed to a trade-off between gluten protein and animal protein. Increasing the concentration of insect flour required decreasing the proportions of wheat, rice and maize flours of the control formulation (Table 1), resulting in a weaker gluten network that affected gas bubbles and water trapping [12]. Using store-bought *T. molitor* and *A. diaperinus* flours with a large particle size caused obstructions in the printer cartridge. Attempts to grind the flour to obtain smaller particles or find cartridges with larger openings were considered but unsuccessful. The solution was to directly purchase dried larvae from the producer and optimize grinding using existing laboratory mills, to achieve a particle size of ±500 μm, allowing the printing process to continue without further issues.

### 3.2. Dough Rheological Properties

Analyzing the rheological properties of the dough-snacks under study reveals a great deal of importance for fine-tuning the printing parameters to achieve successful printing. Success in printing is not only determined by the machine’s ability to place the printable material on a plate, but it is successful when the dough is placed on the plate while exhibiting the intended characteristics in the design. These characteristics include well-defined layers, fine detailing and intricate patterns [40]. All these parameters contribute to a better consumer experience of edible insects in food, particularly in the case of snacks in this study. Therefore, a deep understanding of the behavior of the doughs is required.

**Figure 4:**
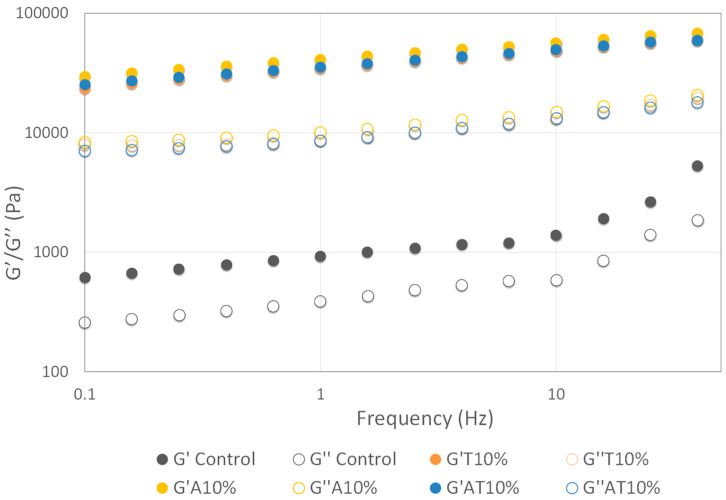
Frequency sweep of the control snack, snack with 10% of *Alphitobius diaperinus* (A10%), snack with 10% of *Tenebrio molitor* (T10%) and snack with 10% of *A. diaperinus* + *T. molitor* (AT10%) samples. G′ (storage modulus)—close symbol; G″ (loss modulus)—open symbol.

**Figure 5:**
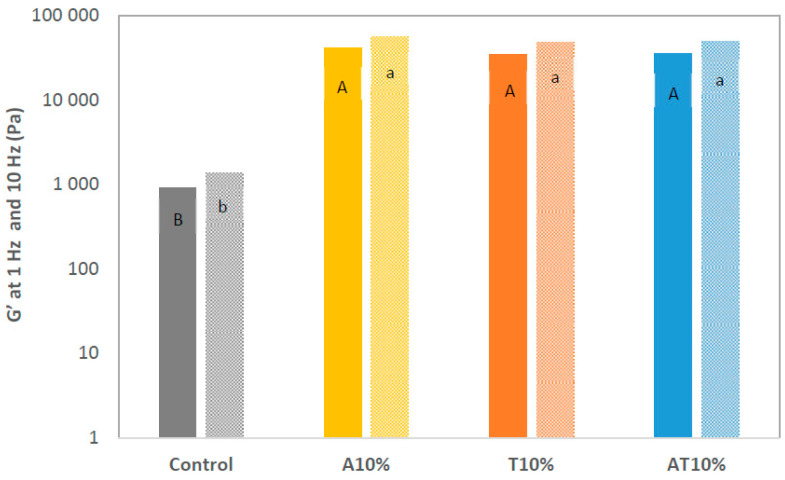
Average value of G′ (storage modulus extracted from the frequency sweep, Pa) at 1 Hz (solid color) and 10 Hz (pattern) of the control snack, snack with 10% of *Alphitobius diaperinus* (A10%), snack with 10% of *Tenebrio molitor* (T10%), and snack with 10% of *A. diaperinus* + *T. molitor* (AT10%) samples. Different letters represent statistically significant differences between groups (*p* < 0.05).

Frequency sweep tests were carried out on the control, T10%, A10% and AT10% samples (mechanical spectra presented in Figure 4). All the samples containing insect flours revealed a high value of G′ and G″, reflecting the presence of a more organized and stable structure, compared to the control. These results are associated with the highest level of detailing observed in the insect-printed samples. Complementing the information given by the frequency sweep analysis, Figure 3 provides the analysis of the average values of the elastic modulus G′ at 1 Hz. The A10% sample exhibited the highest level of internal structuring with an average G′ (1 Hz) of 41,000 ± 16,310 (Pa), a considerably higher value compared to the control sample (923 ± 56 Pa) (Figure 5).

**Figure 6:**
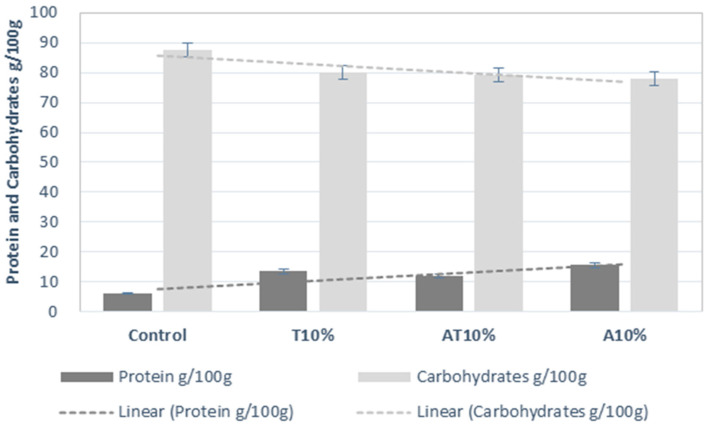
Variation in carbohydrates * and protein (g/100 g, mean values and their respective standard deviations) of the control snack, snack with 10% of *Alphitobius diaperinus* (A10%), snack with 10% of *Tenebrio molitor* (T10%) and snack with 10% of *A. diaperinus + T. molitor* (AT10%) samples. * Calculated by difference.

Figure 5 shows a higher degree of internal structure in the dough when the insect flour is added, as the protein content increases, derived from the amount of protein coming from the insect flours (Table 2). The increase in protein content is followed by a decrease in carbohydrates, as shown in Table 2. This behavior is not observed in the samples containing both *T. molitor* and *A. diaperinus* flours. These samples do not reach high levels of protein nor exhibit sharp decreases in carbohydrates, as can be seen in Figure 6. However, it was also observed that after the 10% concentration was reached, the doughs incorporating only *T. molitor* and *A. diaperinus* showed a loss of internal structure strength once higher concentrations of insect flour were incorporated in the snacks (non-presented results). Thus, a cap of 10% incorporation of insect flour was established for experimental purposes. Comparing the values of G′ (1 Hz) values in the literature, it was observed that they correspond with the printability maps created by Oliveira et al. [28] for 3D printed snacks with the incorporation of microalgae biomass. However, the obtained values are greater than those obtained by Uribe-Wandurraga et al. [41] and simultaneously follow short of the G′ (1 Hz) values obtained by Letras et al. [27] also in snacks with microalgae incorporation and other protein-based doughs [40]. This indicates more work must be done to better understand G′ (1 Hz) variations when creating a printability map. It is important to consider that the works mentioned before focused on 3D printed doughs with no incorporation of insect flour.

### 3.3. Snack Texture

**Figure 7:**
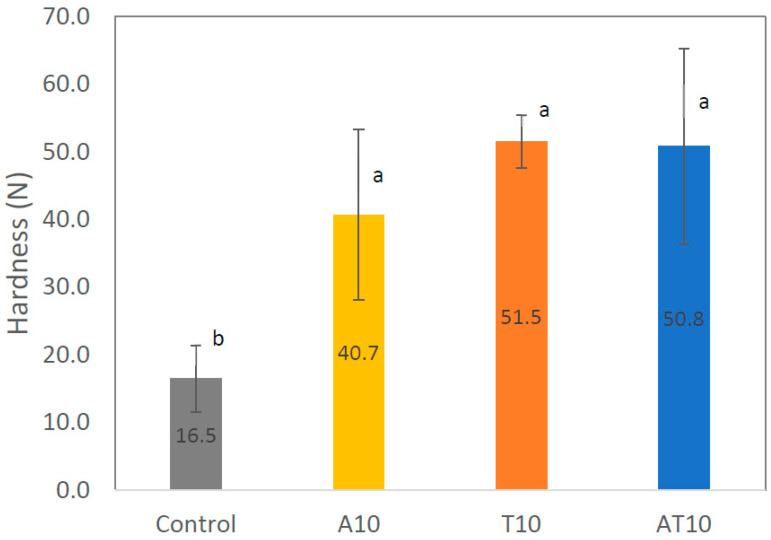
Hardness (N, mean values and their respective standard deviations) of the control snack, snack with 10% of *Alphitobius diaperinus* (A10%), snack with 10% of *Tenebrio molitor* (T10%) and snack with 10% of *A. diaperinus* + *T. molitor* (AT10%) samples. Different letters represent statistically significant differences between groups (*p* < 0.05).

Measurements were conducted to obtain the hardness (Figure 7) of the snacks. The hardness of the insect-snack samples was significantly (*p* ˂ 0.05) higher than the value obtained for the control samples, in accordance with the rheology data. This suggests that the addition of protein through the incorporation of 10% *A. diaperinus* and/or *T. molitor* can harden the snack, although the gluten, which makes a major contribution to the texture of wheat products, is reduced. There were no significantly different results obtained for the brittleness of the snacks. Comparing these hardness values (16.5–51.5 N) with Oliveira et al.’s [28] values for printing snacks with microalgae (0.8–18.1 N), it can be observed that higher values were obtained in the present study, which are not distant from Letras et al.’s [27] hardness values (12.4–39.1 N) measured in 3D snacks enriched with microalgae. However, a different effect was found for the gluten-free snacks with spirulina developed by Letras et al. [27], with spirulina-enriched snacks presenting lower hardness values when compared with the control, despite higher dough viscosity and viscoelasticity. 

### 3.4. Nutritional Characterization

Assessing the nutritional values of the 3D printed snacks after the post-treatment process included evaluating moisture content, mineral content, fat, protein, fiber, carbohydrates (calculated by difference) and energy (kcal), and the results are shown in Table 2. 

The focus was on the formulations with a 10% concentration due to their rheological characteristics and potential acceptance by consumers based on sensory characteristics (previously preliminarily studied with different contents of incorporation).

The incorporation of *T. molitor* and *A. diaperinus* in the snacks resulted in a significant decrease (*p* < 0.05) in moisture content compared to the control sample. This decrease can be attributed to the reduction in protein content derived from gluten, being then replaced by animal protein, weakening the network’s ability to retain gases and water. Additionally, the water retention capacity of insect flour and the cooking process during post-treatment contribute to lower moisture contents. The most notable increase observed was in the protein values of the insect-containing snacks, which significantly exceeded the protein content of the control sample. This aligns with previous studies on the nutritional profile of insects and the development of foods incorporating insect flour [42,43,44].

The insect-containing snacks exhibited a substantial increase in their total fat content when compared to the control sample (Table 2). There was no significant increase in the total dietary fiber. The noticeable decline in carbohydrates is related to the reduction in wheat, rice and maize flours, which was complemented with the incorporation of insect flour.

The A10%, T10% and AT10% snacks containing insect flour, according to Regulation (EC) No 1924/2006 [45], if made with selling intent, could claim to be a “source of” protein as more than 12.5% of the energy provided by the snacks derives from protein alone, as can be observed in Table 2.

The mineral composition of the snacks containing insect flour (Table 3) varied significantly. Studies carried out by Adámková et al. [19] perceived that depending on the feed provided to the insects during their breeding the output of the nutritional content of said insects can vary, although there are not sufficient data to claim the same statement regarding the significant differences between snacks containing *Tenebrio molitor* and *Alphitobius diaperinus*. Among the mineral compositions, sodium exhibited the most noteworthy concentration, which was higher in the insect-containing samples due to the natural presence of salt in the insects. Other minerals such as potassium, magnesium, phosphorus, sulphur, copper and zinc also showed higher percentages compared to the control sample.

The 3D printed snacks that incorporated insect flour demonstrated improvements in several nutritional characteristics, compared to the control sample. The protein content increased significantly, while the fat content showed a moderate increase. In addition, there was a decrease in moisture content and slight variations in fiber and carbohydrate content were observed. The mineral composition of the snacks was influenced by the feed provided to the insects, with notable increases in sodium and other minerals.

### 3.5. Antioxidant Potential and Total Phenolics

By exploring the phenolic compounds in edible insects, and evaluating their bioactive capacity and bioavailability, additional health benefits associated with consuming insects can be uncovered. However, this particular study did not focus on characterizing phenolic compounds, so future research should investigate this aspect. Table 4 presents the results of the antioxidant activity, measured by the DPPH and FRAP methodologies, and the total phenolic content (TPC) of the snack formulations: A10%, T10%, AT10% and the control.

As expected, the control sample, without insect flour, showed no antioxidant activity. In contrast, all samples containing edible insects exhibited significant antioxidant activity, although there were no significant differences. 

The antioxidant activity (expressed as ascorbic acid equivalents mg/g dry weight), determined by FRAP, of the control, A10%, T10% and AT10% snacks is presented in Table 4. Antioxidant activity was present in all formulations including the control, although it was significantly higher in snacks containing insect flours. Regarding DPPH results, the control sample quantified 0.1681 AAE mg/g DW, with a statistically significant difference from the other samples. Analyzing the FRAP results (Table 4), the control sample presented antioxidant activity (0.27 ± 0.01 AAE mg/g dry weight). In addition, the difference between the control and the other three samples is significant (*p* < 0.05), with the sample with the highest antioxidant activity being the one which contains *T. molitor* and *A. diaperinus* flours in a total amount of 10% (0.994 ± 0.105 AAE mg/g dry weight), although there are no significant differences. The antioxidant activity of insects has been previously attributed to the presence of phenolic compounds, mainly flavonoids, which have been recognized to be efficient antioxidants [44]. Regarding the total phenolic compounds (Table 4), as mentioned previously in this work, insects have been shown to carry these types of compounds, primarily by absorbing them from their diet, but also through the process of sclerotization. Sclerotization is an absorption process in which insects harden their cuticle. The incorporation of phenolic compounds into the cuticular matrix, which involves structural proteins and chitin, is accomplished through a series of enzyme-mediated reactions [25,44]. Similar to other compounds absorbed via diet, phenolic compounds can vary depending on the feed given to the insect during their developmental stages [22]. However, the diet of the insects used by these authors was unknown at the moment of writing the present work.

The results showed a significant (*p* < 0.05) difference between the control sample (0.11 ± 0.03 GAE g/g dry weight) (Table 4) and the A10%, T10% and AT10% (0.64 ± 0.08, 0.56 ± 0.09 and 0.59 ± 0.04 GAE g/g dry weight, respectively) samples.

Comparing the results obtained with this study to those of [44] conducted on *Acheta domesticus*, another species authorized for commercialization in the EU, it is evident that the commercially fed *A. domesticus* achieved a phenolic compound concentration of 0.01 ± 0.08 (GAE g/g dry weight). This concentration is even lower than the control results, already disregarded due to potential cross-contamination. A more significant comparison can be made when considering the A10%, T10% and AT10% samples containing edible insects. These results exhibited superior values when compared with the [44] study (0.64 ± 0.08, 0.56 ± 0.09 and 0.59 ± 0.04 GAE g/g dry weight, respectively).

It is important to note that the Folin–Ciocalteu reagent used in the analysis is not specific to phenolic compounds and can also react with other compounds, such as amino acid residues. This introduces uncertainty in the method’s results, as they may indicate the presence of bioactive molecules other than phenolic compounds [44].

### 3.6. Opinion Study

**Figure 8:**
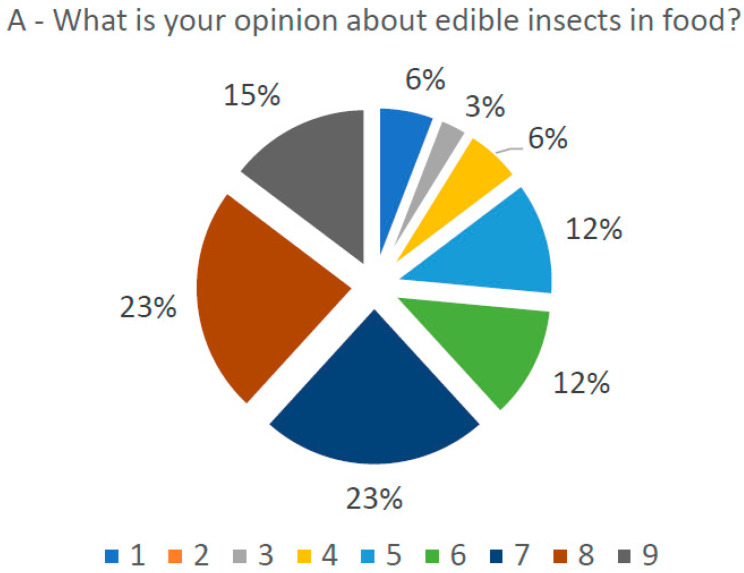

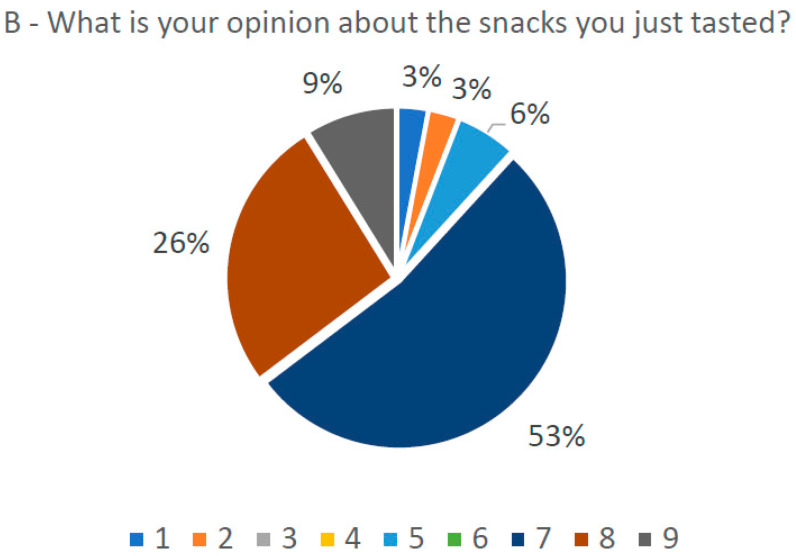
(**A**) General opinion of the panel (N = 34) about the inclusion of insects in food prior to the tasting of the snacks. (**B**) General opinion of the panel (N = 34) about the analyzed snacks after tasting the snacks, using a hedonic scale from 1 to 9.

Using a hedonic scale from 1 to 9, with 1 being the worst and 9 the best, the opinion study revealed that the panelists did express appreciation for snacks enriched with insect flour (Figure 8B), even though 12 panelists demonstrated an opinion lower than 7 about consuming or including insects in their diets (Figure 8A). This demonstrates a shift away from the cultural resistance to entomophagy usually associated with Western consumers [8]. It is possible to see a change in opinion from the first question (Figure 8A) to the last question (Figure 8B) as panelists’ ratings changed from 4, 5 or 6 on the scale before the tasting to 7, 8 or 9 after the taste test. This indicates positive feedback from the panel to the acceptance of insect-based snacks. The general opinion of the panelists about the snacks was very positive, with only one response below 5. The sensory analysis results may be an indication that consumers are more receptive to the introduction of this innovative food with edible insects, combined with the appellative designs provided by the 3D printing process (Figure 8A,B).

## 4. Conclusions

In the present study it was found to be possible to develop insect-based snacks with favorable nutritional profiles, characterized by high alternative animal protein content and relevant concentrations of minerals, antioxidants and phenolic compounds. The use of 3D printing technology proved to be beneficial in creating visually appealing food designs. However, a limitation was observed in the form of nozzle clogging, which resulted from the heterogeneity of the formulations and their tendency to dry at the nozzle end. This study contributes to the exploration of 3D printing applications in the field of food, particularly in innovative food with edible insects. It has potential to promote sustainable, environmentally friendly and healthy dietary habits, while addressing nutrient shortages. Future research should employ GC-MS methodology to investigate antioxidants, phenolic compounds and fatty acids composition, to obtain a further detailed profile. A deeper understanding of the 3D printing process is crucial for innovative designs and introducing new food products to consumers. Long-term studies concerning how to breed and feed insects with different diets can be explored to personalize food and meet individual-specific needs.

## Figures and Tables

**Table 1:** Table of ingredients contained in the control sample (C1), formulation containing 10% of *Alphitobius diaperinus* flour (A10%), formulation containing 10% of *Tenebrio molitor* flour, and formulation containing 10% *Alphitobius diaperinus* (5%) + *Tenebrio molitor* (5%) (AT10%).

Ingredients	Control	A10%	T10%	AT10%
Wheat flour (g)	15.00	12.50	12.50	12.50
Rice flour (g)	15.00	12.50	12.50	12.50
Maize starch (g)	23.80	17.75	17.75	17.75
Xanthan gum (g)	0.20	0.25	0.25	0.25
Salt (g)	1.00	1.00	1.00	1.00
Edible oil (g)	5.00	-	-	-
Insect flour (g)	0.00	10.00	10.00	10.00
Water (g)	40.00	46.00	46.00	46.00
Total weight (g)	100.00	100.00	100.00	100.00

**Table 2:** Nutritional characterization (mean values and their respective standard deviation, ±SD) of moisture, ash, total fat, protein, fiber and carbohydrate contents (%) and energy (kcal) of the control snack, snack with 10% of *Alphitobius diaperinus* (A10%), snack with 10% of *Tenebrio molitor* (T10%) and snack with 10% of *Alphitobius diaperinus* + *Tenebrio molitor* (AT10%) samples. Different letters represent statistically significant differences between groups (*p* < 0.05). * Calculated by difference.

Samples	Control	T10%	A10%	AT10%
Moisture (%)	2.02 ± 0.03 ^a^	0.82 ± 0.01 ^c^	0.36 ± 0.01 ^d^	1.72 ± 0.06 ^b^
Ash (%)	3.12 ± 0.05 ^a^	4.06 ± 0.00 ^b^	4.48 ± 0.01 ^a^	3.43 ± 0.09 ^d^
Total fat (%)	0.04 ± 0.02 ^d^	0.13 ± 0.09 ^c^	0.15 ± 0.04 ^b^	0.23 ± 0.12 ^a^
Protein (%)	6.01 ± 0.47 ^d^	13.46 ± 0.85 ^bc^	15.64 ± 0.85 ^a^	13.35 ± 0.61 ^b^
Fiber (%)	1.25 ± 0.68 ^a^	1.51 ± 0.59 ^a^	1.43 ± 0.23 ^a^	1.99 ± 0.16 ^a^
Carbohydrates (%) *	87.57	80.03	77.94	79.29
Energy (kcal/100 g)	379.69	381.11	381.34	380.58

**Table 3:** Mineral composition (mg/100 g, mean values and their respective standard deviations, ±SD) of the control snack, snack with 10% of *Alphitobius diaperinus* (A10%), snack with 10% of *Tenebrio molitor* (T10%) and snack with 10% of *Alphitobius diaperinus* + *Tenebrio molitor* (AT10%) samples. Standard deviation is displayed with each value. Different letters represent statistically significant differences between groups (*p* < 0.05).

Samples	Control% + SD (mg/100 g)	T10% + SD (mg/100 g)	A10% + SD (mg/100 g)	AT10% + SD (mg/100 g)
Na	657.97 ± 2.61 ^d^	787.81 ± 4.38 ^c^	865.09 ± 12.00 ^b^	1058.34 ± 6.87 ^a^
K	95.02 ± 0.65 ^d^	220.45 ± 4.19 ^c^	235.82 ± 4.30 ^b^	236.01 ± 4.19 ^a^
Ca	14.25 ± 13.71 ^a^	13.48 ± 1.82 ^a^	26.74 ± 1.76 ^a^	18.88 ± 3.51 ^a^
Mg	17.79 ± 0.23 ^d^	55.23 ± 0.60 ^a^	35.22 ± 0.48 ^c^	47.41 ± 0.60 ^b^
P	70.69 ± 2.14 ^d^	188.16 ± 1.46 ^c^	199.19 ± 3.02 ^a^	198.57 ± 1.52 ^b^
S	70.38 ± 1.00 ^d^	111.06 ± 2.05 ^c^	131.20 ± 2.05 ^a^	122.88 ± 1.20 ^b^
Fe	2.59 ± 0.94 ^a^	3.05 ± 0.20 ^a^	2.20 ± 0.25 ^a^	3.47 ± 0.97 ^a^
Cu	0.42 ± 0.01 ^a^	0.70 ± 0.03 ^b^	0.74 ± 0.05 ^b^	0.69 ± 0.02 ^b^
Zn	0.68 ± 0.02 ^d^	2.76 ± 0.02 ^bc^	2.69 ± 0.04 ^b^	2.82 ± 0.03 ^a^
Mn	0.59 ± 0.04 ^a^	0.61 ± 0.01 ^a^	0.56 ± 0.01 ^a^	0.58 ± 0.01 ^a^
B	0.34 ± 0.48 ^a^	0.06 ± 0.02 ^a^	0.05 ± 0.01 ^a^	0.06 ± 0.01 ^a^

**Table 4:** DPPH, FRAP and total phenolic compounds (mean values and their respective standard deviations, ±SD) of the control snack, snack with 10% of *Alphitobius diaperinus* (A10%), snack with 10% of *Tenebrio molitor* (T10%) and snack with 10% of *Alphitobius diaperinus* + *Tenebrio molitor* (AT10%) samples. Different letters represent statistically significant differences between groups (*p* < 0.05).

	DPPH (AAE mg/g DW)	FRAP (AAE mg/g DW)	TPC (GAE g/g DW)
Control	0.17 ± 0.03 ^a^	0.27 ± 0.01 ^a^	0.11 ± 0.03 ^a^
A10%	0.90 ± 0.12 ^b^	0.87 ± 0.07 ^b^	0.64 ± 0.08 ^b^
T10%	0.81 ± 0.04 ^b^	0.92 ± 0.07 ^b^	0.56 ± 0.09 ^b^
AT10%	0.75 ± 0.04 ^b^	0.99 ± 0.10 ^b^	0.59 ± 0.04 ^b^

## Data Availability

The original contributions presented in the study are included in the article, further inquiries can be directed to the corresponding author.
